# Novel inference models for estimation of abundance, survivorship and recruitment in mosquito populations using mark-release-recapture data

**DOI:** 10.1371/journal.pntd.0005682

**Published:** 2017-06-26

**Authors:** Daniel Antunes Maciel Villela, Gabriela de Azambuja Garcia, Rafael Maciel-de-Freitas

**Affiliations:** 1 Programa de Computação Científica, Fundação Oswaldo Cruz, Rio de Janeiro, Brazil; 2 Laboratório de Transmissão de Hematozoários, Instituto Oswaldo Cruz, Fundação Oswaldo Cruz, Rio de Janeiro, Brazil; University of California, Davis, UNITED STATES

## Abstract

**Background:**

Experiments involving mosquito mark-release-recapture (MRR) design are helpful to determine abundance, survival and even recruitment of mosquito populations in the field. Obstacles in mosquito MRR protocols include marking limitations due to small individual size, short lifespan, low efficiency in capturing devices such as traps, and individual removal upon capture. These limitations usually make MRR analysis restricted to only abundance estimation or a combination of abundance and survivorship, and often generate a great degree of uncertainty about the estimations.

**Methodology/Principal findings:**

We present a set of Bayesian biodemographic models designed to fit data from most common mosquito recapture experiments. Using both field data and simulations, we consider model features such as capture efficiency, survival rates, removal of individuals due to capturing, and collection of pupae. These models permit estimation of abundance, survivorship of both marked and unmarked mosquitoes, if different, and recruitment rate. We analyze the accuracy of estimates by varying the number of released individuals, abundance, survivorship, and capture efficiency in multiple simulations. These methods can stand capture efficiencies as low as usually reported but their accuracy depends on the number of released mosquitoes, abundance and survivorship. We also show that gathering pupal counts allows estimating differences in survivorship between released mosquitoes and the unmarked population.

**Conclusion/Significance:**

These models are important both to reduce uncertainty in evaluating MMR experiments and also to help planning future MRR studies.

## Author summary

Mosquito-borne diseases such as dengue and malaria impose a global burden with recurrent outbreaks. Recently, emergence of arboviral diseases caused by Zika and chikungunya viruses has also become a global concern. Knowledge about the ecology of mosquito populations under natural conditions may provide significant aid to help designing more effective vector control strategies. Quantitative metrics such as the abundance of mosquito populations are difficult to be measured in the field without resorting to experiments with markers. There are, however, limitations to these kinds of experiments such as short mosquito lifespan, marking limitations due to small body size, low efficiency in capturing devices such as traps, and once-only individual capture. Due to these limitations most methods estimate either only abundance or a combination of abundance and survivorship. In this work, we present statistical methods designed to estimate abundance, survivorship and recruitment using inference models and information such as counts of pupae. Results indicate that having low capture efficiencies as often observed in field assays still permits good estimation. Also, low number of released mosquitoes compromise density and survival estimations. We expect these methods to be helpful to people collecting mosquito field data and for health analysts to evaluate possible outcomes of control interventions.

## Introduction

Mark-release-recapture (MRR) methods applied to study mosquito populations permit analysis of vector survival, dispersal, and abundance in natural environment. Various mosquito species, in particular of the *Aedes*, *Culex* and *Anopheles* genera, are vectors associated with persistent diseases such as dengue, filariasis and malaria and also emergent infections by chikungunya and Zika viruses. Given such medical importance, early mathematical models for malaria transmission [1,2] established the vectorial capacity as an important metric to assess epidemic risk by a mosquito population. Reliable vectorial capacity assessment requires accurate estimations of mosquito density (mosquitoes/human) and survivorship (daily survival probability). These estimates typically help to improve vector control policies and practices in endemic regions and might lead to mitigation of disease transmission [3].

By their nature, mosquito MRR experiments have important design restrictions that hinder the application of more sophisticated capture-recapture models such as the commonly known Jolly-Seber method [4]. For example: (a) individual mosquitoes are released and typically not recaptured multiple times because once collected at traps they do not survive for new releases, (b) recapture rates are low, often ranging from 5–10% [5], (c) most of the experimental designs, with notable exceptions [6], consider groups of marked individuals as cohorts due to small mosquito body size and consequent difficulty of individual marking methods and because a high number of mosquitoes are released from a few selected points, and (d) average lifespan under natural conditions is short. These limitations restrict models which consider individual markers and multiple recaptures. In early designs of capture-recapture experiments involving mosquitoes, most works used deterministic estimators such as Lincoln-Petersen and Fisher-Ford indexes to evaluate vector abundance [3,7]. Currently, deterministic models are still used mainly due to lower mathematical complexity, when compared to stochastic/Bayesian models. In the case of the Lincoln-Petersen index, the ratio between the number of marked individuals recaptured and the total insects released allows estimation of the total abundance from the count of captures of unmarked individuals. For an MRR experiment spanning at most a dozen days, we have observations over multiple days, but only a low number of recaptures due to low capture efficiencies at traps. In the case of mosquito populations, Lincoln-Petersen abundance estimation is expectedly inaccurate, since the number of marked mosquitoes alive for trapping after a few days is significantly smaller than the number released due to a sharpened mortality across the released cohort, plus trapping on previous days [8–10]. In fact, daily captures of mosquitoes at traps typically exhibit an exponential decay largely due to mortality of marked individuals. The Fisher-Ford model [11] is another deterministic method that requires the probability of daily survival to adjust the capture ratio for the multiple estimations over time. Estimates of survival probability are possible using MRR data from the exponential decay of capture counts of marked individuals. In order to estimate abundance, Buonaccorsi *et al*. [12] consider not only the survival probability but also removal of individuals captured at traps. Recruitment in mosquito MRR experiment areas occurs either through birth or immigration. Recruitment rate estimation is possible under stable abundance, even though still challenging due to mosquito MRR limitations.

Here we build Bayesian models that leverage the concepts behind the Fisher-Ford model [11] and Buonaccorsi *et al*.’s model [12]. Moreover, we propose another two novel Bayesian approaches to estimate relevant parameters of mosquito population biology such as adult population size, survival rates and recruitment. Recruitment estimation is possible if assuming equal adult survival rates or including a component into the model that uses counts of immature individuals, typically pupae. For various mosquito species such as *Ae*. *aegypti* pupae are known to present low mortality and thus are likely to emerge as adult individuals [13]. Analyses using these models permit us to infer abundance, survivorship and recruitment rate using both field data and datasets obtained from simulations, when taking into account counts of immature individuals. Furthermore, our results reveal the degree of tolerance of these methods to both capture efficiency at traps and number of released mosquitoes.

## Materials and methods

### MRR–*Aedes aegypti*

We used the capture counts of adult females obtained from trap collections in the Z-10 neighborhood located at the city of Rio de Janeiro, Brazil, during an MRR experiment described by Villela *et al*. [10]. We used data from experiment ST2, in which a single release point (map available in Villela *et al*. [10]—supplementary files) was considered. We summed the number of trapped individuals over all traps for each day in the study. Before releases started, pupal surveys were carried out over all of the breeding sites found in the 66 premises containing an adult trap, observed in the same occasion when the trap was installed. A total of 212 larvae and 47 pupae were collected in 7 containers from 7 (11%) different dwellings. All immatures were collected in man-made containers such as plastic plant dishes and uncovered water tanks and were brought to the entomology laboratory at Fiocruz for further classification using taxonomic keys. The choice about using number of adult females is due to the use of adult traps specifically designed to attract female mosquitoes [10].

### Ecological processes and experimental design

#### Capturing

Traps used in MRR experiments capture mosquitoes possibly using substances to attract them. Capturing, however, is not perfect as only a subset of all released mosquitoes are collected at traps due to not fully covering the experiment area. Each single trap covers a limited area over which a mosquito can be attracted to it and trapped. Both this capability of being attracted and the probability of being captured, once attracted, are together described quantitatively here as trap capture efficiency *β*_*0*_.

#### Survivorship

We consider survivorship only during the adult stage. There is evidence of senescence in *Aedes aegypti* mosquito, i.e. mortality rate increases with mosquito age [14]. Most models, however, describe survivorship by a single parameter. An age-dependent parameter estimated from laboratory experiments is unlikely to represent field conditions. We consider a constant probability of daily survival (PDS) during adult phase. We consider female survivorship since generally only females are released in MRR experiments and traps are designed to capture females. Factors affecting mortality include predation, lack of resources, harsh climatic conditions and use of chemical compounds. The usual quantitative measurement to describe survivorship is the probability of daily survival *φ* in the case of marked mosquitoes (*φ*_*u*_ for unmarked mosquitoes).

#### Recruitment

Recruitment rate *b* of individuals includes immigration and births. Births are clearly density dependent, whereas immigration might not be. Other factors also impact recruitment such as climate conditions, for instance water resources for breeding sites impacting adult emergence. However, birth rate will not vary significantly within a short duration of an MRR experiment. Concerning immigration, we typically assume that an MRR site is geographically restricted such that any potential flow of new mosquitoes from outside areas is neglected.

#### Abundance

Here abundance *U* is the number of females estimated for the whole area in which a MRR experiment is carried out. This abundance might also be presented as indirect quantity such as a ratio of number of females per premise.

#### Pupal search

Collection of immature individuals may happen before MRR experiment. Since searches are typically imperfect, we describe the efficiency of pupal search by parameter *μ*.

### MRR data for mosquito population—Simulations

Several designs are used for mosquito MRR trials. Guerra *et al*. [15] assembled data from publicly reported mosquito MRR trials and provided a quantitative synthesis. In most of the experimental designs mosquitoes typically are not recaptured multiple times and are marked as cohorts using markers such as fluorescent dust. In MRR experiments, such as reported by Maciel-de-Freitas *et al*. [8,9] and Ritchie *et al*. [16], typically counts of recaptured mosquitoes from all cohorts (signaled by color of fluorescent dust) and counts of unmarked, captured mosquitoes are taken at each of multiple traps across an area over around a 10-day period.

We simulated multiple scenarios numerically (for instance, varying number of releases, capture probabilities and survival probabilities). Each simulation requires initial conditions and parameters such as abundance *U* at the beginning of the experiment, daily probabilities *φ* and *φ*_*u*_ of survival for both marked and unmarked individuals, the daily recruitment rate *b*. An MRR study requires a number *D* of days of mosquito collection at traps. Mosquito capture occurs with a given capture efficiency *β*_*0*_, the number of released mosquitoes *N*, and the number of traps *J*. A pupal search in the experiment area that typically would occur in the field a day or two earlier than releasing time collects a number of pupae *n*_*pupae*_. If the pupal search is imperfect, the number of pupae collected is given by *μn*_*pupae*_, where *μ* describes the efficiency of pupal search. The simulation returns the daily numbers *m*_*i*_ and *u*_*i*_ of individuals captured at traps, both marked and unmarked ones, respectively. Table 1 shows the variables, parameters used in the simulation model and a short description.

**Table 1:** Model variables. List of variables used in the models and their respective descriptions.

Variables	Description
*U*_*i*_	Number of unmarked individuals at time *i*. If abundance is constant over time, the index might be removed.
*N*	Number of marked individuals released in the field.
*φ*	Probability of daily survival for marked individuals.
*φ*_*u*_	Probability of daily survival for unmarked individuals.
*B*	Daily recruitment rate.
*Μ*	Efficiency of pupal search.
*P*	Probability of capture at traps at a day period.
*n*_*pupae*_	Number of pupae collected before the experiment.
*m*_*i*_, ***m***	Number of marked individuals captured at day *i*, vector containing *m*_*i*_
*u*_*i*_,***u***	Number of unmarked individuals captured at day *i*, vector containing *u*_*i*_.
*β*_*0*_	Daily capture efficiency at mosquito traps.
*Τ*	Pupal maturation time
D	Number of collection days in the MRR experiment.
$m_{c}=\sum_{1}^{D}m_{i}$	Total number of marked individuals recaptured in the experiment.
$u_{c}=\sum_{1}^{D}u_{i}$	Total number of unmarked individuals captured in the experiment.

S1 Table describes the used parameters in our simulations and our models. The number of days, number of immatures and number of traps did not vary in the simulations.

### Multinomial Poisson inference

The inference models describe relationships between the known values, such as number *N* of released mosquitoes, the number of days post-release *D*, and observed data, such as numbers *m*_*i*_ and *u*_*i*_ of marked and unmarked mosquitos collected at traps at day *i*, 1 ≤ *i* ≤ *D*, using parameters to be estimated.

Let ***p*** be the vector of probabilities of capture along the observation periods *i*, 1 ≤ *i* ≤ *D*. First, we consider the number of individuals captured over the MRR experiment time as $m_{c}\;\sim \;Binomial\left(\Sigma_{i=1}^{D}p_{i},\;N\right)$. Then, we take a multinomial distribution for the observations *m*_*i*_ captured at each day *i*: ***m*** ~ Multinomial(***p***, *m*_*c*_).

For a first naïve model M_0_, we consider the probability *p*_*i*_ of capture to be only dependent on the trap capture efficiency *β*_0_. For a second model M_S_, we describe the capturing probability by a product of capture efficiency *β*_0_ and time effects, to be estimated. Therefore, log (*p*_*i*_) = *θ*_0_ + *i θ*_1_, where the estimated capture efficiency *β*_0_ = exp(*θ*_0_) and estimated survival probability *φ* = exp(*θ*_1_). Such a model is more general than model M_0_, since the basic assumption in model M_0_ is equivalent to assume simply that *θ*_1_ = 0, which corresponds to no mortality effects at any time *i*. Multiple daily estimations applying models M_0_ and M_S_ are Bayesian counterparts to multiple values of abundance obtained by Lincoln-Petersen and Fisher-Ford estimators, respectively. For a third model M_B_, we allow for removal of individuals given the daily captures at traps in a Bayesian counterpart to the model proposed by Buonaccorsi *et al*. [12]. In this case, the probability of capture is *p*_*i*_ = *β*_0_(1 − *β*_0_)^i−1^*φ*^*i*^, for marked individuals and *p*_*i*_ = *β*_0_(1 − *β*_0_)^i−1^, for unmarked individuals. For unmarked individuals, this model does not permit estimation of probability of daily survival *φ*_*u*_. In this case, the underlying assumption is that over a short period of time, typically few days, recruitment is equal to mortality.

The observed number *u*_*i*_ of unmarked individuals collected at traps is modeled as *u*_*i*_ ~ Poisson(*U p*_*i*_), for models M_0_, M_S_, and M_B_, where the abundance number *U* is to be estimated. We use a prior distribution for abundance *U* ~ *Gamma*(0.001, 0.001). We also have prior distribution for capture efficiency *β*_0_ ~ *Beta*(2,4) and for probability of daily survival of marked individuals *φ* = *Beta*(4,2), which are lightly informative distributions, concentrating most mass at values close to 0 in the case of capture efficiency and close to 1 in the case of probability of daily survival.

### Multinomial Poisson models with recruitment

We build two other models that include a recruitment component, including one considering the number of pupae collected from experiments before releasing mosquitoes. We build these models using relationships also described for model M_B_, *i*.*e*., having survivorship equal along with the experiment days and also accounting for removal of individuals. We also consider for both models the number of individuals captured over the MRR experiment time as $m_{c}\;\sim \;Binomial\left(\Sigma_{i=1}^{D}p_{i},\;N\right)$. Then, we take a multinomial distribution for the observations *m*_*i*_ captured at each day *i*: ***m*** ~ Multinomial(***p***, *m*_*c*_).

We define model M_RSU_ for which we assume survival of unmarked individuals equal to the one of marked individuals, i.e., essentially *φ*_*u*_ = *φ*. Therefore, over a short period of time such as an MRR experiment duration, recruitment should occur at rate that maintains population at a constant level. The number of unmarked individuals at each time period, i.e. at risk of being trapped, is the sum of a number *U*_*i*_ of surviving individuals from start of the experiment and the total number *V*_*i*_ of recruited individuals. In the model a number of mosquitoes given by a recruitment rate *b* enter the experiment at each time interval. Therefore, from time *i-1* to time *i* the number of mosquitoes should increase by rate *b*_*i*_ = b*β*_0_(1 − *β*_0_)^*i*−1^*φ*^*i*^. We have the same vector of probability capture described for model M_B_, *p*_*i*_ = *β*_0_(1 − *β*_0_)^*i*−1^*φ*^*i*^. In model M_RSU_, the sum of remaining individuals is a latent variable given by *U*_*i*_ ~ *Poisson*(*U*(1 − *β*_0_)^*i*−1^*φ*^*i*^) and the number of recruited individuals is another latent variable given by $V_{i}\;\sim \;Poisson\left(b\Sigma_{j=1}^{i}{\left(1-\beta_{0}\right)}^{j-1}\varphi^{j}\right)$.

We define model M_RP_ distinguishing the probabilities of daily survival of unmarked and marked mosquitoes, in order to estimate parameter *φ*_*u*_. We describe the number of immature collected before the experiment to be *n*_*pupae*_ ~ *Binomial*(*f*_*a*_(1 − *φ*_u_),*τ U*/*s*), where *f*_*a*_ is a factor that describes how extensive is the immature search, *τ* is the pupal maturation time and *s* is the fraction of the targeted group in the mosquito population. Very commonly, the purpose is to estimate the abundance of female mosquitoes. Here, pupal maturation time is *τ* = 2 days and the fraction of female mosquitoes is *s* = 0.5 [3]. Factor *f*_*a*_ represents an adjustment since the immature search typically covers only a fraction of the area surveyed, or alternatively a fraction of the number of premises. We have the remaining and recruited individuals assessed in the same way, but survivorship for unmarked individuals is given by *φ*_*u*_: capture counts of surviving individuals $U_{i}\;\sim \;Poisson\left(U\;{\left(1-\alpha \right)}^{i-1}\varphi_{u}{}^{i}\right)$ and recruitment quantities $V_{i}\;\sim \;Poisson\left(b\Sigma_{j=1}^{i}{\left(1-\beta_{0}\right)}^{j-1}\varphi_{u}{}^{j}\right)$.

For both models M_RSU_ and M_RP_, the observed number of individuals is given by *u*_*i*_ ~ *Binomial*(*β*_0_, *U*_*i*_ + *V*_*i*_). We use a prior distribution for abundance *U* ~ *Gamma*(0.001, 0.001), for capture efficiency *β*_0_ ~ *Beta*(2,4) and for probability of daily survival of individuals *φ* = *Beta*(4,2) and *φ*_*u*_ = *Beta*(4,2), where appropriate, and for basic recruitment rate *b* ~ *Lognormal*(10, 0.25).

As a reference, Table 2 describes the assumptions behind each of these models and which estimators can be extracted from them.

**Table 2:** Description of models M_0_, M_S_, M_B_, M_RSU_, and M_RP_. Models are built using observed data and different assumptions. Depending on observed data, each model permits distinct parameters to be estimated. Some of these models are closely related to other methods proposed in the literature as shown in the counterpart model column.

Bayesian models	Description					Estimation
	Number of recaptures	Survivorship	Removal of individuals	Number of pupae	Counterpart model	
M_0_	Yes	-	-	-	Lincoln-Petersen estimator	Abundance,
M_S_	Yes	Yes	-	-	Fisher- Ford estimator [11]	Abundance, survival
M_B_	Yes	Yes	Yes	-	Buonaccorsi et al. [12]	Abundance, survivorship
M_RSU_	Yes	Yes	Yes	-	-	Abundance, survivorship, recruitment
M_RP_	Yes	Yes	Yes	Yes	-	Abundance, survivorship (marked and unmarked), recruitment

### Computational platform

We implemented the simulation tool using the R platform [17]. We also wrote description models using the WinBUGS language [18] for the statistical models (M_0_, M_S_, M_B_, M_RSU_, M_RP_). We analyze the simulation data via Monte-Carlo Markov chain simulations (MCMC), by running 3 separate chains, 360,000 iterations during each of the chains, with a 320,000 burn-in period. These numbers sufficed for good convergence except otherwise noted within our results. We use R to load the simulation data and streamline pre-processed data via package R2JAGS [19] into JAGS [20], the selected tool for MCMC analysis. Output from MCMC analysis permits us to obtain samples of the posterior distribution, and as a result, mean and median values, as well as credibility intervals (CI). In S1 Text we present boxes that contain the description of our models prepared for JAGS tool. Our scripts for simulation and analysis are publicly available at https://github.com/DVMath/MosqCapRecap.

## Results

### Estimation of abundance, survivorship and recruitment using field data

**Fig 1:**
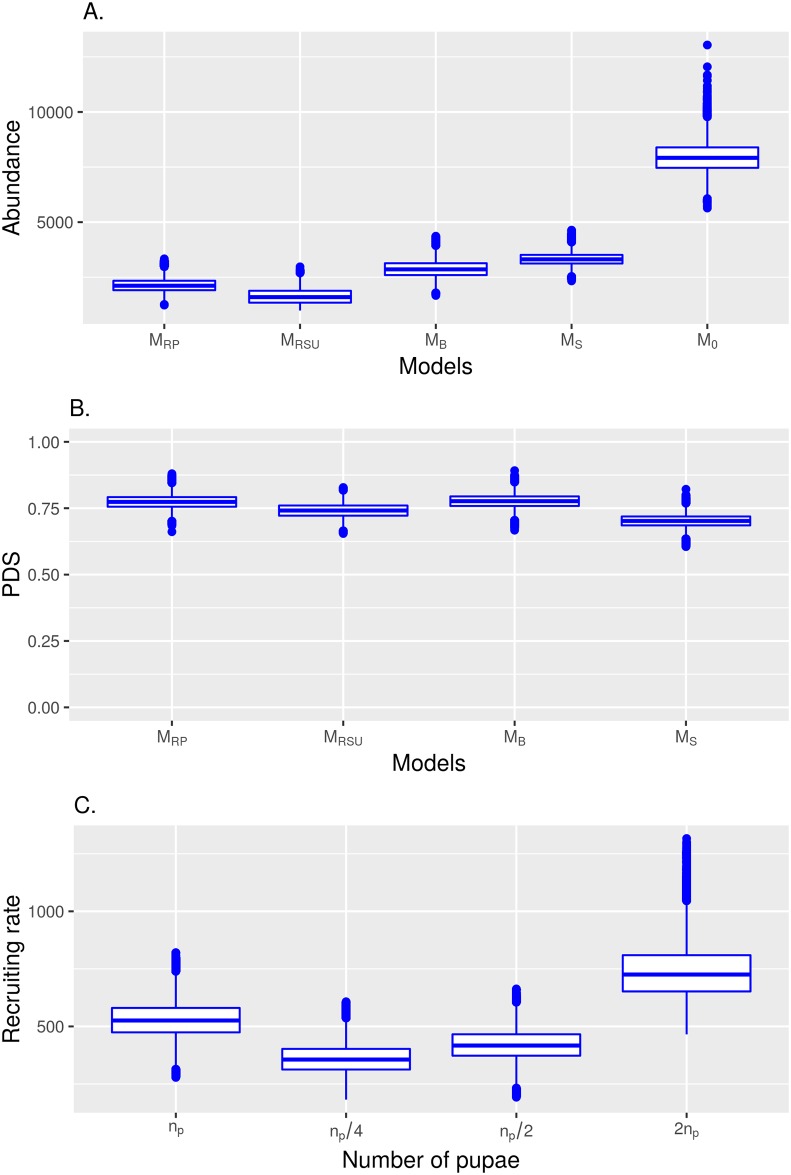
Estimation of abundance, survivorship, and recruiting rate in a study area in the city of Rio de Janeiro, Brazil. (A) Abundance of mosquitoes (number of females in the Z-10 area). (B) Probability of daily survival. (C) Recruiting rate, where *n*_*pupae*_ is the number of pupae collected. Outlier values are shown by points.

We estimated abundance of *Aedes aegypti* mosquito population in the Z-10 neighborhood in Rio de Janeiro from inference analysis using models M_0_, M_S_, M_B_, M_RSU_, and M_RP_. Results from using model M_0_ reveal a much larger abundance (Fig 1A). Indeed, an overestimation is expected, since this model does not consider either survival estimation or removal of individuals. Estimation from the posterior distribution results average values of abundance in the releasing day that are 3,326 (95% CI: 2,794–3,944), 2,875 (95% CI: 2,171–3,676), 1,636 (95% CI: 1,152–2,345), and 2,143 (95% CI: 1,574–2,890) female mosquitoes, from analysis using models M_S_, M_B_, M_RSU_, M_RP_, respectively. Probability of daily survival from posterior distributions obtained from analyses of models M_B_, M_RSU_, and M_RP_ were very similar (Fig 1B). In the case of model M_RP_ the mean probability of daily survival was 0.77 (95% CI: 0.72–0.83). The mean recruiting rate was estimated at 530 mosquitoes per day (95% CI: 383–701) for model M_RP_. Since the method is sensitive to the number of pupae collected in the field, we estimate abundance using model M_RP_ considering various alternative possibilities such as a twofold, half and a quarter of the collected number of pupae (Fig 1C). The last two possibilities (half and quarter of the collected number) result in smaller abundance estimations, when considering the collected number to be the closest to the number of pupae in the area. By contrast, a larger recruiting rate is expected if the real number of pupae to be collected is twofold.

### Estimation of abundance, survivorship and recruitment using simulated datasets

Table 3 contains results obtained from multiple simulation experiments using models M_0_, M_S_, M_B_, M_RSU_, M_RP_. Model M_RP_ included the assumed input values of abundance within credibility intervals in 16 simulation studies (indicated by the number of asterisks in the M_RP_ column). For assumed values of abundance of 8,000 mosquitoes and above, model M_RP_ underestimated the abundance. For values of probability of daily survival of unmarked individuals less than 0.8, model M_RP_ resulted in either overestimation (study 19) or underestimation (studies 23 and 25).

**Table 3:** Abundance estimates from simulated data. The simulation study number refers to the study identifier (S1 Table). Results (means and credibility intervals) are shown at thousands for clarity purposes. An asterisk (*) indicates whether the credibility interval contains the assumed abundance value (shown at first column).

Simulation Study	Abundance (input value)	Abundance mean value estimates at thousands (cred. intervals)				
		M_RP_	M_RSU_	M_B_	M_S_	M_0_
1	4,000	4.2 (3.6–5.1)*	3.6 (3.0–4.2)*	3.4 (2.8–4.1)*	2.5 (2.0–2.9)	8.4 (7.6–9.3)
2	4,000	3.3 (2.5–4.3)*	2.4 (1.9–3.1)	2.7 (1.9–3.7)	1.5 (1.1–2.0)	5.8 (5.0–6.7)
3	4,000	3.9 (3.2–4.9)*	3.1 (2.6–3.7)	3.1 (2.4–3.7)	2.1 (1.7–2.6)	7.0 (6.2–7.9)
4	4,000	4.4 (3.5–5.5)*	3.2 (2.6–3.9)	3.9 (3.0–4.8)*	2.6 (2.1–3.1)	7.8 (6.9–8.8)
5	4,000	3.8 (3.3–4.3)*	3.5 (3.0–4.1)*	3.0 (2.5–3.4)	2.3 (2.0–2.7)	8.7 (7.9–9.5)
6	4,000	4.2 (3.6–4.8)*	3.7 (3.2–4.3)*	3.5 (3.0–4.0)*	2.8 (2.4–3.2)	9.1 (8.4–9.9)
7	2,000	2.3 (2.0–2.7)*	1.9 (1.6–2.3)*	2.2 (1.9–2.7)*	1.7 (1.4–2.1)*	6.9 (6.2–7.7)
8	8,000	6.5 (5.5–7.7)	6.1 (5.3–7.0)	4.6 (3.8–5.5)	3.3 (2.8–3.9)	11.7 (10.7–12.8)
9	6,000	5.3 (4.5–6.2)*	4.7 (4.0–5.4)	4.1 (3.4–4.9)	3.0 (2.5–3.5)	10.1 (9.2–11.1)
10	4,000	3.5 (2.9–4.1)*	3.0 (2.5–3.6)	2.6 (2.1–3.1)	2.0 (1.7–2.3)	7.1 (6.4–7.9)
11	4,000	2.7 (1.9–4.1)*	2.1 (1.5–2.8)	2.2 (1.3–3.3)	1.0 (0.7–1.5)	4.6 (3.8–5.5)*
12	4,000	3.9 (2.8–5.3)*	2.4 (1.8–3.1)	3.2 (2.2–4.8)*	1.6 (1.2–2.2)	4.8 (4.1–5.7)
13	4,000	3.2 (2.3–4.4)*	2.5 (1.9–3.2)	2.3 (1.6–3.3)	1.4 (1.0–1.8)	5.7 (4.9–6.7)
14	10,000	8.6 (7.1–10.0)	7.4 (6.5–8.4)	5.5 (4.6–6.6)	4.2 (3.6–4.8)	12.0 (11.0–13.1)
15	4,000	4.0 (3.2–4.9)*	3.3 (2.7–3.9)	3.4 (2.7–4.3)*	2.4 (2.0–2.9)	8.1 (7.3–9.1)
16	4,000	3.7 (3.3–4.3)*	3.6 (3.2–4.1)*	2.7 (2.3–3.1)	2.1 (1.8–2.4)	8.6 (7.9–9.4)
17	4,000	4.6 (4.0–5.2)*	4.4 (3.8–4.9)*	3.4 (3.0–3.9)	2.6 (2.3–3.0)	10.1 (9.2–10.9)
18	4,000	3.6 (3.1–4.3)*	3.0 (2.5–3.5)	2.9 (2.4–3.5)	2.2 (1.8–2.6)	6.6 (6.0–7.3)
19	4,000	4.8 (4.1–5.5)	3.4 (2.9–4.0)	3.1 (2.6–3.6)	2.4 (2.0–2.8)	5.5 (5.0–6.1)
20	12,000	9.4 (8.0–10.7)	9.0 (8.0–10.1)	6.2 (5.1–7.3)	4.6 (4.0–5.4)	14.6 (13.4–16.0)
21	14,000	10.8 (9.5–12.2)	9.9 (8.8–11.2)	7.4 (6.1–9.0)	5.3 (4.6–6.2)	16.1 (14.8–17.5)
22	4,000	3.8 (3.3–4.4)*	3.3 (2.8–3.8)	2.8 (2.4–3.3)	2.3 (2.0–2.6)	6.9 (6.3–7.5)
23	4,000	3.5 (3.0–3.9)	3.2 (2.8–3.6)	2.6 (2.3–3.0)	2.2 (1.9–2.5)	7.1 (6.6–7.8)
24	4,000	4.2 (3.6–4.8)*	3.5 (3.0–4.0)	2.9 (2.5–3.4)	2.4 (2.0–2.7)	5.8 (5.3–6.4)
25	4,000	3.5 (3.0–3.9)	3.0 (2.6–3.4)	2.4 (2.1–2.7)	2.0 (1.8–2.3)	5.8 (5.3–6.3)

Comparing assumed input values for study 1 and its estimations in Table 4, all parameters were estimated close to the assumed values and the 95% credibility intervals indeed contain these assumed values. Analysis by model M_RP_ results in abundance of 4,220 mosquitoes (95% CI: 3,572–5,067) for an assumed abundance value of 4,000 mosquitoes. We also estimated probability of daily survival (PDS) for unmarked at 0.86 and marked individuals at 0.77 and recruitment rate 624 individuals/day. Analysis from model M_RSU_ reveals an estimation of a 95% credibility interval also containing the abundance value for simulation study 1. The probability of daily survival, however, is wrongly estimated due to the assumption of equal survival rates for all individuals whether marked or not. Model M_B_ permits estimation of abundance, probability of daily survival (marked individuals) and trap capture efficiency. Estimates given by model M_B_ are also close to the assumed values, which are well within the 95% credibility intervals. Model M_S_ does not consider removal of individuals, an assumption that proves costly since it underestimated both the abundance and probability of daily survival. Model M_0_ results greatly overestimate abundance due to not considering the daily survival.

**Table 4:** Results for all parameters from analysis via MCMC using the described models and simulation dataset # 1. Simulated data were obtained using parameter values in the first line (Input value). Results from analysis running MCMC simulations (3 chains, 360,000 iterations, 320,000 burn-in period) are shown in the subsequent lines (Estimation). Mean values and credibility intervals (95%) are obtained from posterior output samples. An asterisk (*) indicates whether the credibility interval contains the assumed input value in the simulation. Parameters not estimated due to the model limitations are signaled by a single dash (-).

Input values	Abundance	Trap capture efficiency	PDS (marked)	PDS (unmarked)	Recruitment (per day)
	*N* = 4,000	*β*_*0*_ = 0.05	*φ* = 0.78	*φ*_*u*_ = 0.85	*b* = 600
Estimation					
M_RP_	4,220 (3,572–5,067)*	0.049 (0.041–0.59)*	0.77 (.75-.81)*	0.86 (0.83–0.88)*	624 (503–760)*
M_RSU_	3,573 (3,013–4,218)*	0.06 (0.05–0.07)*	0.75 (0.72–0.78)*	0.75 (0.72–0.78)	993 (865–1,140)
M_B_	3,394 (2,805–4,086)*	0.05 (0.04–0.06)*	0.78 (0.75–0.81)*	-	-
M_S_	2,458 (2,048–2,926)	0.06 (0.05–0.08)*	0.72 (.69–0.75)	-	-
M_0_	8,402 (7,599–9,305)	0.018 (0.016–0.020)	-	-	-

### Number of releases impact estimation

**Fig 2:**
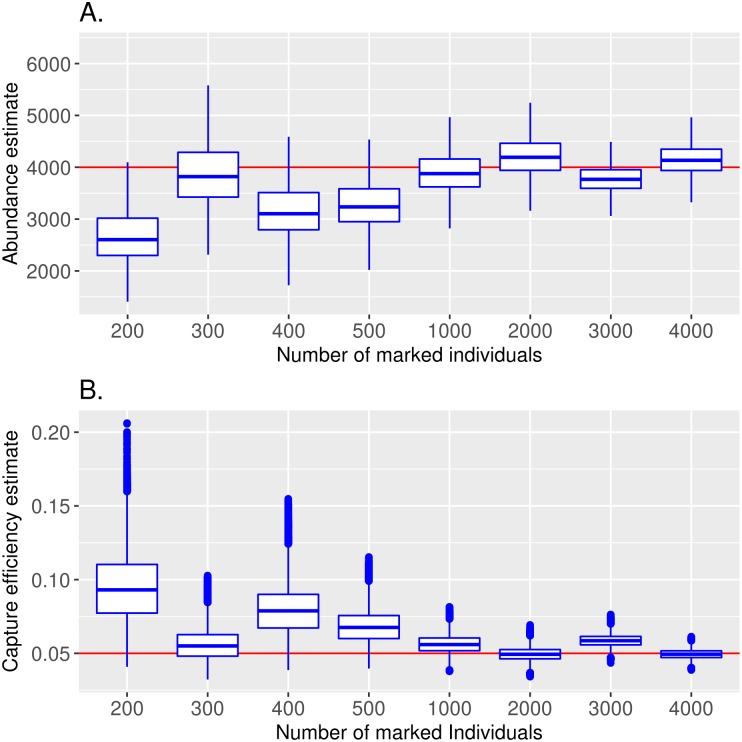
Impact of release numbers on parameter estimates. Results are shown for the posterior distributions (mean and 95% credibility intervals) of abundance (A) and capture effciency (B). Horizontal lines indicate the assumed input value for simulation. Points indicate outliers. Released numbers less than 1,000 reveal either mean not close to the assumed value or large 95% credibility interval/poor convergence.

For simulations with at least 1000 marked mosquitoes, mean estimated abundance values are close to the assumed values, which are within the 95% credibility interval. Values below 1,000 marked mosquitoes were not quite as close to the estimation value. Also, the 95% credibility interval in these cases gets much larger as size of the released cohort decreases. Inspection of results from very low values indicates high uncertainty, as expected (Fig 2A). Fig 2B indicates that the low levels of capture counts due to relatively few release numbers prove costly to the capture efficiency estimation resulting in overestimation. As a consequence, abundance is underestimated.

### Distinguishing survival from unmarked to marked individuals

**Fig 3:**
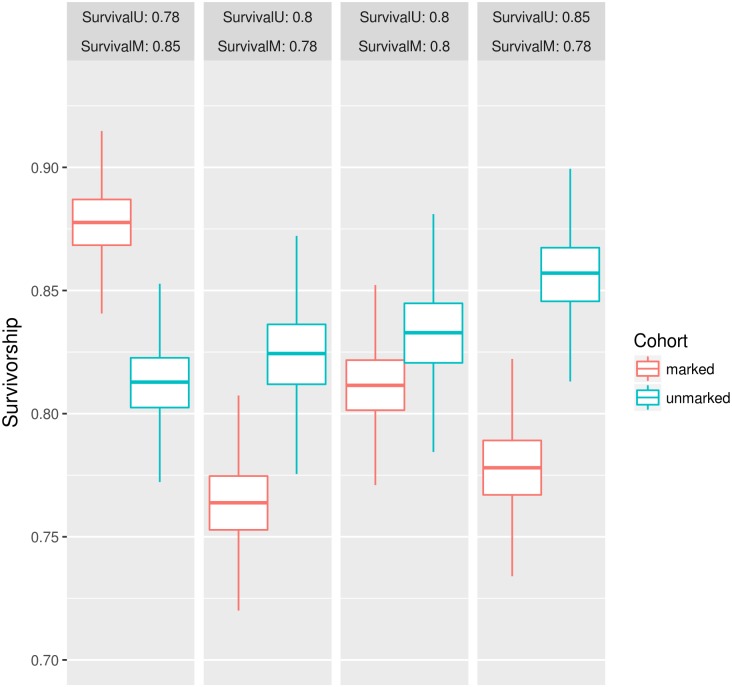
Estimates of probability of daily survival under M_RP_ model under distinct conditons. All simulation parameters are equal at all experiments, except for varying probability of daily survival. Abundance is 4,000 mosquitoes and 2,000 marked mosquitoes are released. Trap capture efficiency is fixed at 0.05. Results are shown for the posterior distributions (mean and 95% credibility intervals). SurvivalU indicates assumed values used for unmarked PDS, whereas SurvivalM indicates assumed values for marked PDS. Red and blue boxplots represent results for marked and unmarked cohorts, respectively.

Fig 3 shows results for survival probabilities under M_RP_ model considering only simulation experiments with same abundance values and release numbers, but varying probability of daily survival of marked individuals and unmarked population. Estimation of PDS for both marked cohort and unmarked cohorts are close to the input values assumed in the simulations, although in some cases for marked population the assumed values are closer to the extremes of the 95% credibility intervals.

### Model with proper number of immatures permits accurate abundance estimates

**Fig 4:**
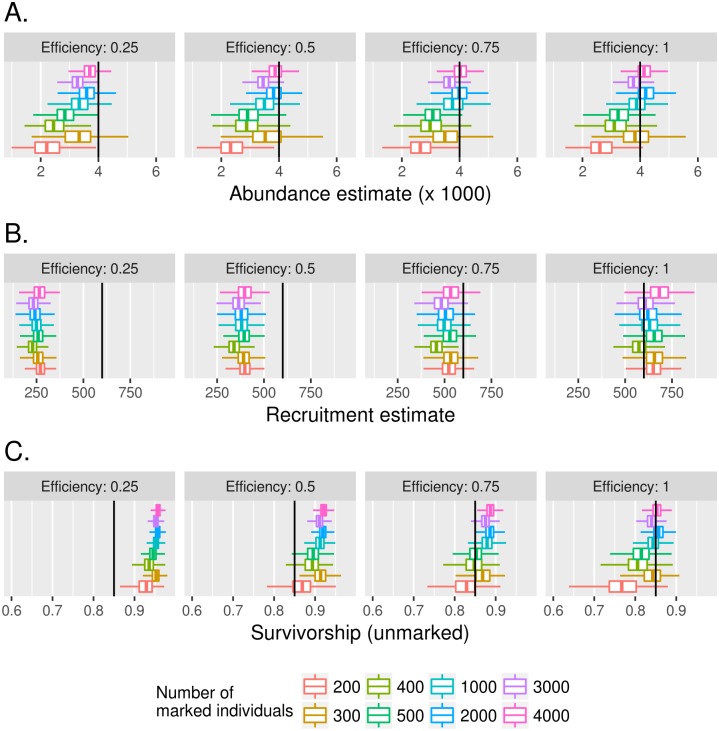
The effect of pupae search efficiency at observing immature counts under M_RP_ model. Here, efficiency describes how good from 0 to 1 counting the immature individuals (pupae) in the pre-MRR phase. Results are shown for the posterior distributions (mean and 95% credibility intervals). Chart A shows abundance results. Chart B indicates recruitment estimates. Chart C indicates survivorship of unmarked individuals. All estimations are intertwined and lowering efficiency causes all of them to deviate from assumed values (black bars). Colors represent the number of released individuals as shown in legend.

If efficiency at collecting pupae is low, results from using model M_RP_ indicate estimations deviating from the assumed input values for abundance, recruitment and probability of daily survival. Fig 4 shows this pupal search efficiency at different levels equal to 25%, 50%, 75% and 100%. As expected, the ideal case (100%) is the best scenario, since estimations are close to the assumed values. For low number of released mosquitoes the estimation also gets worse due to the low capture counts.

### Estimating recruitment assuming equal probability of survival among marked and unmarked individuals

**Fig 5:**
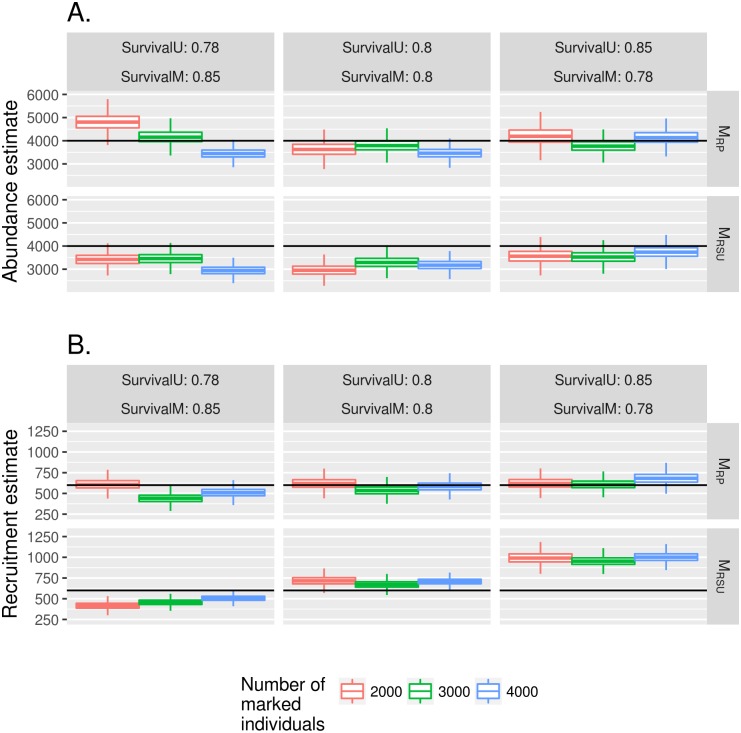
Distinct values of survival rates between marked population and unmarked population impact in abundance and recruitment estimates under models M_RSU_ and M_RP_. Results are shown for the posterior distributions (mean and 95% credibility intervals). Colors represent the number of released individuals. When the absolute value of the difference between survival of unmarked and marked individuals is 0.07, estimates of either abundance (A) or recruitment and (B) are not close to the assumed value.

Fig 5 shows the impact of distinct probabilities of daily survival between marked individuals and unmarked individuals. The cases in the middle column correspond to the assumption in the model, and assumed input values in the simulations lie within the 95% credibility intervals. In the cases where there is difference (left and right columns) between the survival of the two populations, results for recruitment rate (Chart B) in model M_RSU_ are not as close to the expected values. Since estimation of all parameters is intertwined, abundance estimates (Chart A) also get worse.

### Capture efficiency impacts estimation

**Fig 6:**
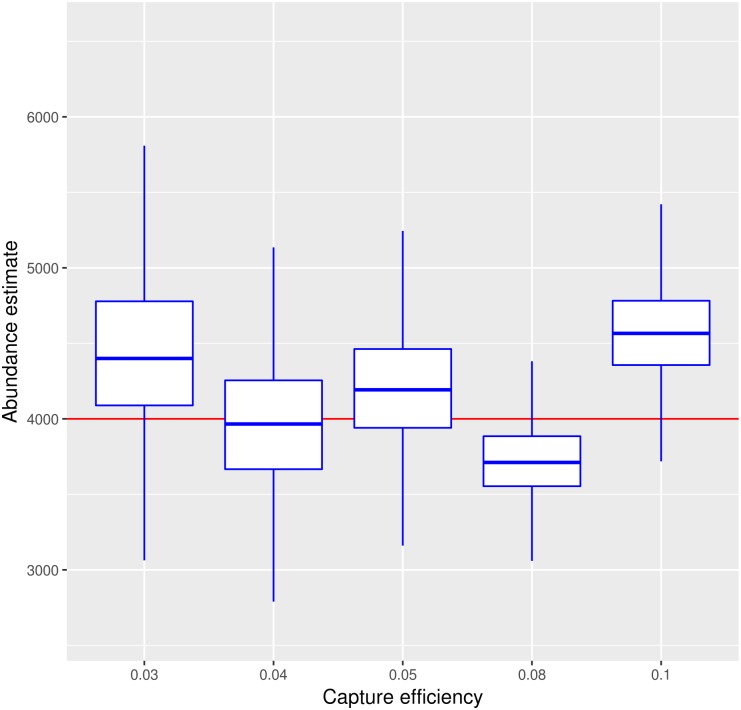
Capture efficiency at traps and its effect in the abundance estimates under M_RP_ model. Capture efficiency varies from values 0.03 to 0.1. Results are shown for the posterior distributions (mean and 95% credibility intervals). All other parameters (abundance, released numbers, recrutiment, survival) were equal across simulation experiments. Abundance is 4,000 mosquitoes and 2,000 mosquitoes are released. Recruitment rate is at 600 mosquitoes/day. Probabilities of daily survival is 0.85 for unmarked cohort and 0.78 for marked cohort. Surprisingly, high capture efficiency does not decrease the 95% credibility intervals significantly.

In Fig 6 capture efficiency varies from 0.03 to 0.1, as we consider only simulation studies that assumed all other parameters (abundance, survival, recruitment) equal. As expected, as the capture efficiency lowers, uncertainty increases, since capture counts are low. As a consequence, 95% credibility intervals are large for capture efficiency smaller than 0.05 (5%). As a surprising effect, the estimations for capture efficiencies at 0.05, 0.08 and 0.1 do not reveal significant difference in their 95% credibility intervals.

## Discussion

We defined Bayesian models to estimate abundance, recruitment and probability of daily survival of mosquito populations in the field from MRR experiment data. Analyses using these models result in posterior distributions for these parameters, hence mean and 95% credibility intervals can be obtained. Moreover, counts from pupal surveys were instrumental to obtain estimated recruitment rates of the wild population. These estimates are particularly interesting since immature counting in breeding sites is one of the most common vector control approaches in countries endemic for arboviruses infections.

Our first set of simple Bayesian inference models is based on estimating the capture efficiency and the probability of daily survival with close relationship to existing methods used for MRR analysis. Since mosquitoes are not often individually captured multiple times (once captured they are effectively removed from the study), a Bayesian model should better describe removal of individuals not only due to mortality but also from the capture process itself. This model has close association to the method proposed by Buonaccorsi *et al*.[12]. Simpler models are defined by neglecting removal of individuals, but still assuming limited survivorship and also neglecting mortality in order to establish Bayesian counterpart models to commonly used Fisher-Ford and Lincoln-Petersen estimators [3,11]. In the case of Lincoln, such approach is not unprecedented since Gaskell and George [21] presented a Bayesian estimation for the Lincoln index. The Bayesian method enabled by our model M_B_ permits inference about the capture efficiency and the probability of daily survival. However, it may not achieve accurate estimations, depending on conditions of large difference between probability survival of marked and unmarked mosquitoes, large abundance or low capture efficiency.

Estimation of recruitment becomes challenging due to the usual mosquito MRR limitations. The concept of using pupal counts for assessment of abundance has been proposed by Focks *et al*. [22] and also advised in other works [15,23]. The estimation implicitly assumes, based on strong sampling efficiency, that pupae numbers should balance with mortality rates, for constant population sizes, therefore the pupae count is the product of the abundance and the mortality rate, but also accounting for sex ratio and the average pupating time. Since our Bayesian framework assumes priors for probability of daily survival and abundance, a description in the model for the number of pupae relating to both survival and abundance is natural, accounting for a factor that the pupae collection might not cover the whole study area. Models M_RP_ and M_RSU_ also permit to estimate recruitment, either assuming collection of pupae or not, respectively. Depending on this information, we can evaluate any potential difference between daily survival of marked and unmarked mosquitoes.

We estimated abundance, survivorship and recruitment rate of an *Aedes aegypti* population in an area in Rio de Janeiro, Brazil, from an experiment conducted in March 2013, described by Villela *et al*.[10]. The mean number per premise varied from 2.1 mosquitoes per premise (*M*_*RSU*_ model) to 4.2 mosquitoes per premise (*M*_*S*_). Such twofold increase shows the importance of choosing the appropriate model to describe parameters of *Aedes aegypti* biology. As shown when using simulated datasets, analysis using model M_RP_ achieves intervals that include the simulation input value in most of the studied scenarios. Daily recruitment rate in the field was about 0.67 mosquitoes per premise in the analysis from model M_RP_. In this case, the recruited number would be about a quarter of the total abundance. The effectiveness of vector control approaches such as targeting the most productive container or using chemical compounds (insecticides) might be evaluated based on potential changes on mosquito recruitment rates. For more effective the vector control intervention, greater decrease in recruitment rate would be expected.

Our results from analyses of simulated datasets show that these models can tolerate capture efficiencies as low as the ones observed for mosquito MRR. We also varied the abundance levels, as opposed to the released numbers, and differences in the survivorship between marked, released mosquitoes and the unmarked population. In the case of immature counts (pupae), recruitment rate can also be estimated, but we find it to be highly dependent on extensive pupal collection, which can require extensive resources in the field.

Limitations in the design of mosquito MRR studies expectedly impact estimation of abundance, survivorship and recruitment rates. First, when abundance is large, the number of released mosquitoes is critical, regardless of the method used. Also capture efficiency in regular MRR experiments is usually small, varying in the range of 5–10% [5,10]. We have shown that such rates are still acceptable, but capture efficiencies below this range lead to higher degree of uncertainty in the estimation. By contrast, to reduce credibility intervals most likely we would need a combination of higher efficiencies and multiple individual recaptures, which is very difficult to implement in the field due to trap conditions. Also, if adapting these methods to have spatial estimations, we expect effects from low capture counts, as opposed to aggregate counts. Otherwise, methods such as proposed by Villela *et al*. [10] that also involve a likelihood component are required due to distance from mosquito concentration areas to traps.

Collecting pupae in the field can be difficult due to limited accessibility to breeding sites, but we think that results from model M_RP_ should motivate getting such samples to have better estimates. Our results indicate sensitivity of recruiting rate estimates when assuming different number of pupae. Because pupal surveys may have difficult feasibility to be conducted on the routine of vector control programs, our results demonstrate that surveys with varying degrees of imperfection lead to biased estimations of abundance, recruitment and survival rates. Conversely, public health decision makers might adopt models such as M_RP_ and M_RSU_ with attention to these issues. For example, the Brazilian dengue national control program recommends a survey 4–6 times yearly in around 10% of cities of each district of important cities to determine infestation and Breteau Indexes, plus the most productive container type across the country [24]. If at least one of these surveys, e.g. the one immediately before dengue transmission starts, has high-quality pupal surveys being conducted in blocks representatives of disease transmission over the city, estimates on vector abundance, survivorship and recruitment rate might be helpful to improve vector control efficiency by directing existing strategies towards areas in which *Ae*. *aegypti* population has greater vectorial capacity.

Our models estimating recruitment assume that the population stays constant during the short period of experiment time. If such assumption does not hold due to abundance fluctuations occurring as a result of changing environmental conditions, use of insecticides, or any other, we expect difficulties to get accurate estimations applying this modeling, unless the exogenous conditions can be modeled.

Simulated datasets and analyses consider typical designs used for MRR experiments involving mosquito populations of *Aedes aegypti*, a known vector of Zika, dengue and chikungunya viruses. However, these models can possibly be applied to other mosquito populations. Laboratory-reared individuals of *Aedes aegypti* used in previous field studies [8–10] had the genetic background of field mosquitoes. In this case, such designs would not necessarily imply different survival of the released mosquitoes compared to the field mosquitoes. However, daily survival estimates are essential for use of modified mosquitoes such as *Wolbachia*-carrying mosquitoes as described by Garcia *et al*. [5].

There is vast literature on MRR experiments to study ecology of wild animal species [25] (and references therein), instead of mosquito populations. Studies with mosquito MRR may benefit from more advanced techniques, including possibility of using covariates such as environmental variables, individual tagging, positional and distance effect, if overcoming important design limitations. For instance, individual marking, multiple sightings and geoposition recording has been done for estimating abundance of mammals [26]. For a few insect populations, individual marking is possible through code systems, such as applying distinct dots to the body [27], for instance by elytra puncture in beetles [28]. Krebs et al. describe density estimation of rodent population in a Canadian area, by live-trapping individuals [29]. More refined models departing from other study designs and including other variables can take elements from capture-recapture designs for populations other than mosquito ones.

Bayesian models permit us to include all parameters instead of serial parameter estimation and to use prior beliefs, if any, or vague priors in order to obtain not only mean estimations but also credibility intervals. Traditional methods require a sequence of estimations for survival and abundance, and if possible recruitment, from observed field data. Smith and McKenzie [30] demonstrated the impact on vector control strategies relying on each of the parameters of the basic reproduction number for the Ross-Macdonald model [1] for malaria. More recently the classical models for malaria have been revisited to study sensitivity in applying strategies for disease control [31,32]. Models for transmissibility of other vector-borne diseases such as Zika, dengue, and chikungunya viruses can also benefit from sensitivity analysis, if using estimated parameters describing the interaction of these pathogens and their vectors. Bayesian models reveal uncertainties that coupled with sensitivities model greatly enhances estimation of vectorial capacities. Advancing towards Bayesian models that encompass a whole set of parameters greatly enhances understanding not only of the underlying dynamics but also on sensitivity to each of the biological aspects. Such models are useful to advance on strategies of vector control that aim at reducing the vectorial capacity of mosquito populations.

## Supporting information

S1 Table Description of simulation parameters for each of the simulation studies. All experiments used a recruitment rate *b* = 600, the number of traps is *J* = 64, and the experiment time is *D* = 10 days.
(DOCX)

S1 Text Bayesian models defined for use in R and JAGS. (DOCX)

## References

[1] Macdonald G. The epidemiology and control of malaria. London: Oxford University Press; 1957.

[2] Garrett-Jones C. The human blood index of malaria vectors in relation to epidemiological assessment. Bull World Health Organ. 1964;30: 241 14153413 PMC2554803

[3] Silver JB. Mosquito ecology: field sampling methods. 3^rd^ ed New York: Springer; 2008.

[4] Seber GAF. A note on the multiple-recapture census. Biometrika. 1965;52: 249–260. 14341277

[5] de Garcia GA, dos Santos LMB, Villela DAM, Maciel-de-Freitas R. Using *Wolbachia* releases to estimate *Aedes aegypti* (Diptera: Culicidae) population size and survival. PLoS One. 2016;11: e0160196 doi: 10.1371/journal.pone.0160196 27479050 10.1371/journal.pone.0160196 PMC4968812

[6] Trpis M, Hausermann W. Dispersal and other population parameters of *Aedes aegypti* in an African village and their possible significance in epidemiology of vector-borne diseases. Am J Trop Med Hyg. 1986;35: 1263–1279. 3789275 10.4269/ajtmh.1986.35.1263

[7] Costantini C, Li S-G, Torre AD, Sagnon N, Coluzzi M, Taylor CE. Density, survival and dispersal of *Anopheles gambiae* complex mosquitoes in a West African Sudan savanna village. Med Vet Entomol. 1996;10: 203–219. 8887330 10.1111/j.1365-2915.1996.tb00733.x

[8] Maciel-de-Freitas R, Codeco C, Lourenço-de-Oliveira R. Body size-associated survival and dispersal rates of *Aedes aegypti* in Rio de Janeiro. Med Vet Entomol. 2007;21: 284–292. doi: 10.1111/j.1365-2915.2007.00694.x 17897370 10.1111/j.1365-2915.2007.00694.x

[9] Maciel-De-Freitas R, Codeco CT, Lourenco-de-Oliveira R. Daily survival rates and dispersal of *Aedes aegypti* females in Rio de Janeiro, Brazil. Am J Trop Med Hyg. 2007;76: 659–665. 17426166

[10] Villela DAM, Codeço CT, Figueiredo F, Garcia GA, Maciel-de-Freitas R, Struchiner CJ. A Bayesian hierarchical model for estimation of abundance and spatial density of *Aedes aegypti*. PLoS One. 2015;10: e0123794 doi: 10.1371/journal.pone.0123794 25906323 10.1371/journal.pone.0123794 PMC4408040

[11] Dowdeswell W, Fisher R, Ford E. The quantitative study of populations in the Lepidoptera *I*. *Polyommatus icarus* rott. Ann Eugen. 1940;10: 123–136.

[12] Buonaccorsi JP, Harrington LC, Edman JD. Estimation and comparison of mosquito survival rates with release-recapture-removal data. J Med Entomol. 2003;40: 6–17. 12597647 10.1603/0022-2585-40.1.6

[13] Focks DA, Sackett SR, Bailey DL, Dame DA. Observations on container-breeding mosquitoes in New Orleans, Louisiana, with an estimate of the population density of *Aedes Aegypti* (L.). Am J Trop Med Hyg. 1981;30: 1329–1335. 7325289 10.4269/ajtmh.1981.30.1329

[14] Styer LM, Carey JR, Wang J-L, Scott TW. Mosquitoes do senesce: departure from the paradigm of constant mortality. Am J Trop Med Hyg. 2007;76: 111–117. 17255238 PMC2408870

[15] Guerra CA, Reiner RC, Perkins T, Lindsay SW, Midega JT, Brady OJ, et al A global assembly of adult female mosquito mark-release-recapture data to inform the control of mosquito-borne pathogens. Parasit Vectors. 2014;7: 276 doi: 10.1186/1756-3305-7-276 24946878 10.1186/1756-3305-7-276 PMC4067626

[16] Ritchie SA, Montgomery LB, Hoffmann AA. Novel estimates of Aedes *aegypti* (Diptera: Culicidae) population size and adult survival based on *Wolbachia* releases. J Med Entomol. 2013;50: 624–631. 23802459 10.1603/me12201

[17] R Core Team. R: A language and environment for statistical computing. Vienna, Austria; 2014 URL http://www.R-Proj.Org. 2015;

[18] Lunn DJ, Thomas A, Best N, Spiegelhalter D. WinBUGS-a Bayesian modelling framework: concepts, structure, and extensibility. Stat Comput. 2000;10: 325–337.

[19] Su Y-S, Yajima M. R2jags: A package for running jags from R. R package version 003–08 URL HttpCRAN R-Proj Orgpackage R2jags. 2012;

[20] Plummer M. JAGS: A program for analysis of Bayesian graphical models using Gibbs sampling. Proceedings of the 3rd international workshop on distributed statistical computing. Technische Universit at Wien; 2003. p. 125.

[21] Gaskell TJ, George BJ. A Bayesian modification of the Lincoln Index. J Appl Ecol. 1972;9: 377–384. doi: 10.2307/2402438

[22] Focks DA, Chadee DD. Pupal survey: an epidemiologically significant surveillance method for *Aedes aegypti*: an example using data from Trinidad. Am J Trop Med Hyg. 1997;56: 159–167. 9080874 10.4269/ajtmh.1997.56.159

[23] Reisen WK, Mahmood F, Azra K. *Anopheles culicifacies* giles: Adult ecological parameters measured in rural punjab province, pakistan using capture-mark-release-recapture and dissection methods, with comparative observations on *An*. *stephensi* liston and *An*. *subpictus* grassi. Res Popul Ecol. 1981;23: 39–60. doi: 10.1007/BF02514092

[24] Coelho GE, Burattini MN, da Teixeira MG, Coutinho FAB, Massad E. Dynamics of the 2006/2007 dengue outbreak in Brazil. Mem Inst Oswaldo Cruz. 2008;103: 535–539. 18949321 10.1590/s0074-02762008000600004

[25] MacKenzie DI, editor. Occupancy estimation and modeling: inferring patterns and dynamics of species. Amsterdam ; Boston: Elsevier; 2006.

[26] Royle JA, Karanth KU, Gopalaswamy AM, Kumar NS. Bayesian inference in camera trapping studies for a class of spatial capture-recapture models. Ecology. 2009;90: 3233–3244. 19967878 10.1890/08-1481.1

[27] Conway GR, Trpis M, McClelland GAH. Population parameters of the mosquito *Aedes aegypti* (L.) estimated by mark-release-recapture in a suburban habitat in Tanzania. J Anim Ecol. 1974;43: 289 doi: 10.2307/3366

[28] Unruh TR, Chauvin RL. Elytral punctures: a rapid, reliable method for marking Colorado potato beetle. Can Entomol. 1993;125: 55–63. doi: 10.4039/Ent12555-1

[29] Krebs CJ, Boonstra R, Gilbert S, Reid D, Kenney AJ, Hofer EJ. Density estimation for small mammals from livetrapping grids: rodents in northern Canada. J Mammal. 2011;92: 974–981. doi: 10.1644/10-MAMM-A-313.1

[30] Smith DL, McKenzie FE. Statics and dynamics of malaria infection in *Anopheles* mosquitoes. Malar J. 2004;3: 13 doi: 10.1186/1475-2875-3-13 15180900 10.1186/1475-2875-3-13 PMC449722

[31] Brady OJ, Godfray HCJ, Tatem AJ, Gething PW, Cohen JM, McKenzie FE, et al Adult vector control, mosquito ecology and malaria transmission. Int Health. 2015;7: 121–129. doi: 10.1093/inthealth/ihv010 25733562 10.1093/inthealth/ihv010 PMC4357799

[32] Brady OJ, Godfray HCJ, Tatem AJ, Gething PW, Cohen JM, McKenzie FE, et al Vectorial capacity and vector control: reconsidering sensitivity to parameters for malaria elimination. Trans R Soc Trop Med Hyg. 2016;110: 107–117. doi: 10.1093/trstmh/trv113 26822603 10.1093/trstmh/trv113 PMC4731004

