# Supplementary material for: Novel inference models for estimation of abundance, survivorship and recruitment in mosquito populations using mark-release-recapture data
Source: PLoS Negl Trop Dis. 2017 Jun 26;11(6):e0005682. doi: 10.1371/journal.pntd.0005682 (PMC5501687; doi:10.1371/journal.pntd.0005682)
Supplement: S1 Table — All experiments used a recruitment rate b = 600, the number of traps is J = 64, and the experiment time is D = 10 days. (DOCX) [file pntd.0005682.s001.docx]

**S1 Table**

**Description of parameters that varied over all simulation scenarios.**

| Simulation |  |  | Capture |  |  |
| --- | --- | --- | --- | --- | --- |
| Experiment | Abundance | Releases | Efficiency | PDS (Marked) | PDS (Unmarked) |
| 1 | 4000 | 2000 | 0.05 | 0.78 | 0.85 |
| 2 | 4000 | 500 | 0.05 | 0.78 | 0.85 |
| 3 | 4000 | 1000 | 0.05 | 0.78 | 0.85 |
| 4 | 4000 | 2000 | 0.03 | 0.78 | 0.85 |
| 5 | 4000 | 3000 | 0.05 | 0.78 | 0.85 |
| 6 | 4000 | 4000 | 0.05 | 0.78 | 0.85 |
| 7 | 2000 | 2000 | 0.05 | 0.78 | 0.85 |
| 8 | 8000 | 2000 | 0.05 | 0.78 | 0.85 |
| 9 | 6000 | 2000 | 0.05 | 0.78 | 0.85 |
| 10 | 4000 | 2000 | 0.05 | 0.78 | 0.8 |
| 11 | 4000 | 200 | 0.05 | 0.78 | 0.85 |
| 12 | 4000 | 300 | 0.05 | 0.78 | 0.85 |
| 13 | 4000 | 400 | 0.05 | 0.78 | 0.85 |
| 14 | 10000 | 2000 | 0.05 | 0.78 | 0.85 |
| 15 | 4000 | 2000 | 0.04 | 0.78 | 0.85 |
| 16 | 4000 | 2000 | 0.08 | 0.78 | 0.85 |
| 17 | 4000 | 2000 | 0.1 | 0.78 | 0.85 |
| 18 | 4000 | 2000 | 0.05 | 0.8 | 0.8 |
| 19 | 4000 | 2000 | 0.05 | 0.85 | 0.78 |
| 20 | 12000 | 2000 | 0.05 | 0.78 | 0.85 |
| 21 | 14000 | 2000 | 0.05 | 0.78 | 0.85 |
| 22 | 4000 | 3000 | 0.05 | 0.8 | 0.8 |
| 23 | 4000 | 4000 | 0.05 | 0.8 | 0.8 |
| 24 | 4000 | 3000 | 0.05 | 0.85 | 0.78 |
| 25 | 4000 | 4000 | 0.05 | 0.85 | 0.78 |
