# Supplementary material for: Novel inference models for estimation of abundance, survivorship and recruitment in mosquito populations using mark-release-recapture data
Source: PLoS Negl Trop Dis. 2017 Jun 26;11(6):e0005682. doi: 10.1371/journal.pntd.0005682 (PMC5501687; doi:10.1371/journal.pntd.0005682)
Supplement: S1 Text — (DOCX) [file pntd.0005682.s002.docx]

**S1 Text**

**Bayesian Models defined for use in R and JAGS**

Models defined in the paper are described here in language appropriate for use in the JAGS tool. Code for reproducing analysis is publicly available from Github: https://github.com/DVMath/MosqCapRecap

**Model M_0_**

model {

for (t in 1:totT) {

X[t] <- p

Xu[t] <- p

}

theta <- sum(X[])

thetau <- sum(Xu[])

Ncapt ~ dpois(theta * N)

Ucapt ~ dpois(thetau * U)

Yu ~ dmulti(Xu, Ucapt)

Y ~ dmulti(X, Ncapt)

U ~ dpois(v)

v ~ dgamma(0.01, 0.01)

p ~ dbeta(2,4)

}

**Model M_S_**

model {

for (t in 1:totT) {

X[t] <- pow(phi,t)*p

Yu[t] ~ dbin(p, U)

}

theta <- sum(X[])

Ncapt ~ dpois(theta* N)

Y ~ dmulti(X, Ncapt)

U ~ dpois(v)

v ~ dgamma(0.01, 0.01)

phi ~ dbeta(4,2)

p ~ dbeta(2,4)

}

**Model M_B_**

model {

for (t in 1:totT) {

X[t] <- pow(phi,t)*pow(1-p, t-1)*p

}

Xu[1] <- 1

for (t in 2:totT) {

Xu[t] <- 1-p*Xu[t-1]

}

theta <- sum(X[])

thetau <- sum(Xu[])

Ncapt ~ dpois(theta* N)

for (i in 1:totT) {

Ut[i] ~ dpois(U*Xu[i])

}

Y ~ dmulti(X, Ncapt)

for (i in 1:totT) {

Yu[i] ~ dbin(p, Ut[i])

}

#priors

Nu ~ dgamma(0.001, 0.001)

p ~ dbeta(2,2)

phi ~ dbeta(4,2)

U <- Nu

}

**Model M_RSU_**

model {

for (t in 1:totT) {

X[t] <- pow(phi,t)*pow(1-p, t-1)*p

# for unmarked p is counted below

Xu[t] <- pow(phi,t)*pow(1-p, t-1)

}

Ru[1] <- phi

for (t in 2:totT) {

Ru[t] <- Ru[t-1] + pow(phi,t)*pow(1-p,t-1)

}

theta <- sum(X[])

thetau <- sum(Xu[])

Ncapt ~ dpois(theta* N)

for (i in 1:totT) {

Ut[i] ~ dpois(Nu*Xu[i])

}

Y ~ dmulti(X, Ncapt)

for (i in 1:totT) {

bt[i] ~ dpois(l*Ru[i])

}

for (i in 1:totT) {

Yu[i] ~ dbin(p, Ut[i]+bt[i])

}

#priors

Nu ~ dgamma(0.01, 0.01)

p ~ dbeta(2,2)

phi ~ dbeta(4,2)

#probability of not being observed

l ~ dlnorm(10, 1/4.0)

U <- Nu+l

}

**Model M_RP_**

model {

for (t in 1:totT) {

X[t] <- pow(phi,t)*pow(1-p, t-1)*p

# for unmarked p is counted below

Xu[t] <- pow(phiu,t)*pow(1-p, t-1)

}

Ru[1] <- phiu

for (t in 2:totT) {

Ru[t] <- Ru[t-1] + pow(phiu,t)*pow(1-p,t-1)

}

theta <- sum(X[])

thetau <- sum(Xu[])

Ncapt ~ dpois(theta* N)

for (i in 1:totT) {

Ut[i] ~ dpois(Nu*Xu[i])

}

Y ~ dmulti(X, Ncapt)

for (i in 1:totT) {

bt[i] ~ dpois(l*Ru[i])

}

for (i in 1:totT) {

Yu[i] ~ dbin(p, Ut[i]+bt[i])

}

#priors

Nu ~ dgamma(0.001, 0.001)

p ~ dbeta(2,2)

phi ~ dbeta(4,2)

phiu ~ dbeta(2,2)

totpup ~ dbin(1-phiu, round(4*U*fa))

#probability of not being observed

l ~ dlnorm(10, 1/4.0)

U <- Nu+l

}
